# Cancer and Syria in conflict: a systematic review

**DOI:** 10.1186/s12885-024-13256-9

**Published:** 2024-12-18

**Authors:** Lena Basha, Hamza Ahmed, Mohamed Hamze, Amaar Awais Ali, Fares Alahdab, Manar Marzouk, Richard Sullivan, Aula Abbara

**Affiliations:** 1 Syrian American Medical Society, Washington, DC, US; 2 Imperial College London, London, UK; 3 Department of Biomedical Informatics, Biostatistics, Epidemiology, Department of Cardiology, University of Missouri, Missouri, US; 4 London School of Tropical Medicine and Hygiene, London, UK; 5 Syria Public Health Network, Southwark, UK; 6 Department of Infection, Imperial College, St Mary’s Hospital, W2 1NY London, UK

**Keywords:** Cancer, Health services, Military conflict, Oncology, Syria, Systematic review

## Abstract

**Background:**

Armed conflict can disrupt oncology care profoundly, resulting in worsened health outcomes for cancer patients. Syria has endured armed conflict for over a decade, resulting in a severe breakdown of its healthcare services. The aim of this systematic review was to assess the available evidence on the burden of cancer and oncology services in Syria and how they have been affected during the conflict.

**Methods:**

Eight academic and six grey literature databases were searched for English- and Arabic-language studies published from March 2011 until February 2024. Studies reporting any outcomes relating to the burden of cancer or the state of oncology services in wartime Syria were considered for inclusion, although case reports and scoping reviews were excluded. A narrative synthesis of findings was performed.

**Results:**

Of 5,801 studies identified, 28 studies from academic (23) and grey literature (5) were eligible. Papers which reported on the burden of cancer showed an overall increase in cancer incidence and mortality between 2012 and 2022 (most recent data available). Most services were noted to be concentrated in Syria’s capital, Damascus. The main identified themes related to the challenges of providing oncology services with staff shortages, chemotherapeutic drug shortages, insufficient radiotherapy services, and a lack of screening and palliative care noted.

**Conclusion:**

There is insufficient high-quality exploration of the burden of cancer and the state of oncology services across Syria in academic and grey literature. Syria’s health system is divided, creating disparities in access to oncology services, most of which are concentrated in Damascus, exacerbating pre-existing inequalities. The sparsity of robust data reinforces the need for high-quality data, including the use of national or other cancer registries with data from all regions of Syria, including those under opposition control. As the country rebuilds its healthcare systems, policymakers should focus on addressing inequities in oncology service availability to support equity of services.

**Supplementary Information:**

The online version contains supplementary material available at 10.1186/s12885-024-13256-9.

## Background

With the rapidly growing and ageing populations worldwide, the global burden of non-communicable diseases (NCDs), including cancer, has increased [1]. Cancer is a leading cause of mortality, with almost 10 million deaths internationally in 2022 alone [2]. The GLOBOCAN (Global Cancer Incidence, Mortality and Prevalence) 2020 study, conducted by the International Agency for Research on Cancer (IARC), suggested that cancer was the first or second leading cause of premature death in almost 100 countries [3]. As the global population will likely rise above 9 billion by 2040 [4], IARC also predicted cancer incidence and mortality to increase in this timeframe by 49.7% and 62.5%, respectively [5, 6].

Providing oncology services and ensuring pathways to care remain open can be particularly challenging during armed conflicts [7]. Contemporary conflict using high grade ordnance creates serious direct and reverberating impacts on complex cancer services forcing the displacement of healthcare workers and patients [8]. Conflict’s effects on social determinants are such that patients present with more advanced disease and challenges around continued healthcare access can also impact continuity of care; these may include delays to starting treatment, missed diseases, inadequate quality or availability of therapeutics which may have a different side effect profile than more recent therapeutics [9, 10]. A recent paper which explored ‘financial toxicity’ among cancer patients in northwest Syria (NWS) noted the complex financial challenges which cancer patients face and that an improved approach to funding in humanitarian contexts is required [11]. Additionally, transport or accommodation costs may also impede access to oncology services, particularly where they are not locally available.

Over thirteen years have passed since the March 2011 Syrian uprising, which subsequently descended into a protracted armed conflict causing the worst humanitarian crisis of the 21st century. Intense fighting has left over 500,000 Syrians killed [12] and displaced over 11 million people from their homes, including 6.7 million internally displaced people (IDPs), as of 2024 [13]. The ongoing conflict has resulted in severe degradation of Syria’s oncology services, exacerbating issues around access to services. Healthcare in Syria has been ‘weaponized’, forcing many healthcare workers (including oncologists and haematologists) to leave Syria [8, 14, 15]. Healthcare access has also been exacerbated by the economic collapse which has left over 90% of Syrians living below the poverty line, with internally displaced people particularly affected [16, 17]. Dramatically worse social determinants coupled with reduced availability of cancer services has affected timely access to oncology services. One study which included breast cancer patients at a hospital in Damascus from 2019 to 2022 found that 61% of patients were diagnosed at stage III or above [18]. Data from England (a health system unaffected by conflict, ) notes that 5-year survival for breast cancer falls from ~ 100% at stage I to ~ 70% and ~ 25% at stages III and IV respectively according to Cancer Research UK [19].

Prior to the conflict, Syria had experienced an epidemiological shift from communicable to non-communicable diseases, with NCDs causing over 75% of total mortality and cancer alone accounting for 8% [20]. The Syrian National Cancer Registry had been set up in 2002 and was a hospital based registry that mainly collected data from hospital records as well as some data from pathology laboratories, with the aim of becoming a reliable source of population based cancer data. Cancers were classified using the International Classification of Disease-Oncology (ICD-O) classification system. It reported a cancer prevalence of 67 per 100,000 population in 2005 [85] and 81 per 100,000 population in 2009 [86]. However, there was limited data availability in 30% of the country and correct reporting of cancer deaths was found to be an issue particularly in rural areas [86]. By 2007 support from WHO IARC became more erratic, impacting overall levels of data collection and uprisings then descent into conflict in 2011 resulted in the complete halt of the registry.

Before the conflict, specialised oncology services were available in Syria, with government-funded cancer clinics providing free care in some areas and a large cancer specialist hospital located in rural Damascus [15]. In the early 2000s there were incremental improvements in oncology care, due to support by international development agencies such as the European Investment Bank and the German GIZ, such as the opening of two new cancer centres in Aleppo and Homs and the introduction of a breast screening program [23]. However, there were also geographical disparities in service provision particularly along urban/rural divides. Healthcare in areas such as Idlib and northeast Syria suffered from low investment, chronic under-staffing and governmental negligence, which have worsened during the conflict [21, 22]. According to the World Health Organisation (WHO), in 2016, some five years into the conflict, there were four hospitals providing cancer care in Damascus city, one in As-Sweida, four in Homs city, three in Tartous, one in Lattakia, one in Hama and three in Aleppo city providing cancer care for the entire population of the north of Syria [23].

Although there has been increasing literature reporting on the burden of cancer (BOC) and the challenges of cancer care for Syrian refugees, there has been no systematic assessment. Published research on Syrian refugees notes multiple challenges leading to delayed diagnosis and a high burden of cost, often borne by the refugees. Kutluk et al. found that 40% of 268 patients reviewed in their study from Konya, Turkey had metastatic disease at presentation [10]. In a Lebanese study of 113 women with breast cancer in Lebanon, 65.5% presented with locally advanced or metastatic disease prompting a worse outcome. A qualitative study by Marzouk et al. explored cancer care for Syrian refugees in Jordan noting the impact of funding, poor access to screening or timely diagnosis on the experience of patients with cancer [24].

This systematic review aims to identify available literature that describes the BOC and the provision of oncology services in Syria to identify gaps in literature and services.

## Methods

This systematic review has been conducted in accordance with the Preferred Reporting Items for Systematic Reviews and Meta-analyses (PRISMA) guidelines [25]. The research questions of this review were:

1. What is described in the academic and grey literature about the burden of cancer during the conflict in Syria?
2. What is described in the academic and grey literature about the quality and availability of oncology services in wartime Syria?

A systematic literature search was conducted up to February 2024 through eight English language databases (Embase, Global Health, Medline, Web of Science, Scopus, LILACS (Latin American and Caribbean Health Sciences Literature), CINAHL (Cumulative Index to Nursing and Allied Health Literature), PsycInfo). A search of the grey literature was also performed through relevant Syrian and international organisational websites (e.g., WHO, UN (united Nations), UNOCHA (United Nations Office for the Coordination of Humanitarian Affairs), UNICEF (United Nations International Children’s Emergency Fund)) and through six grey literature databases (ReliefWeb, Google Scholar, Health Management Information Centre King’s, OpenGrey, NICE (National Institute for Health and Care Excellence) Evidence Search).

### Inclusion criteria

Since we expected a narrow evidence base, we chose to expand the search by using broad inclusion criteria. Populations of interest were people of all ages and ethnicities located within Syria at the time of recording. We included all papers published between March 2011 and February 2024. Reported outcomes had to be relevant to one of two broad themes: (i) BOC or (ii) provision of oncology services. For oncology provision, any paper addressing the quality of services or Penchansky & Thomas’s five dimensions of healthcare access was deemed relevant [26]. Academic studies could be of any mixed methods, quantitative or qualitative study design, but case reports and scoping reviews were excluded. Grey literature included published reports or factsheets from international organisations, but newspaper articles were excluded. Only the most recently published report was included if reports were part of a series. Table 1 shows the inclusion and exclusion criteria of this study.

**Table 1:** A table showing the inclusion and exclusion criteria for the systematic review

	Inclusion Criteria	Exclusion Criteria
**Geographical Location**	Syrian Arab Republic	Syrian refugees outside of the borders of the Syrian Arab Republic
**Population**	Oncology patients of all age groups. Can be of any ethnicity/refugee/citizenship status in Syria’s borders	None
**Study Period**	March 2011-February 2024	Data only before March 2011 or after February 2024
**Reported Outcome**	Qualitative/quantitative data on the burden of cancer or the availability/quality of oncology services	
**Study Designs**	Mixed methods/ quantitative/ qualitative study designs from academic or grey literature	Case reports, scoping reviews Grey – Data not published in the form of an official report/ fact sheet e.g. newspaper articles
**Data Source Type**	Primary and secondary data sources including cohort reviews, case-controlled studies.	Literature reviews
**Language**	English and Arabic language articles	Non-English or -Arabic language

### Search strategy

Searches were conducted on academic databases using search terms falling under two themes, (i) location (e.g., Syria) and (ii) cancer (e.g., oncology). To ensure the inclusion of all relevant literature, search strings included truncated versions of search terms, spelling variants, misspellings, synonyms, and medical subject heading (MeSH) terms where possible. Table 2 shows an overview of the search strategy.

**Table 2:** An overview of the search strategy used for academic databases

Search No.	Search Term
#1	Syria OR Syrian Arab Republic
#2	Eastern Mediterranean
#3	Levant
#4	Damascus OR Aleppo OR Daraa OR Deir ez-Zor OR Hama OR Homs OR Lattakia OR Raqqa OR Al-Hasakah OR Qamishli OR Tartus OR Douma OR Ghouta OR Manbij OR Idlib OR Quneitra OR As-Suwayda
#5	#1 **OR** #2 **OR** #3 **OR** #4
#6	Neoplasm
#7	Carcinoma
#8	Tumour
#9	Oncology
#10	Cancer
#11	#6 **OR** #7 **OR** #8 **OR** #9 **OR** #10
#12	#5 **AND** #11

Where appropriate, MeSH terms, spelling variations and truncations of each search term were also included in searches

### Study selection

Three researchers screened the search results (HA, Aal and LB); where there was a discrepancy, two other researchers mediated (AAb and MH.) Citations of studies from search results were imported into Covidence, a systematic review management software. Title and abstract screening were performed, followed by full-text screening by three researchers (HA, Aal and LB). The included papers from these two screening phases moved forward to the data extraction phase.

### Data extraction

Key information from each paper was extracted onto a prepared data extraction spreadsheet. Columns were arranged to record important variables of interest. Column headings fell under three main categories:

1. Source Identifiers – ‘First Author’, ‘Publication Year’, ‘Study Title’.
2. Source Characteristics – ‘Study Design’, ‘Study Setting’, ‘Study Period’, ‘Population’.
3. Findings – including ‘Methods’, ‘Key Findings’, ‘Conclusions’ and ‘Limitations’. The ‘Key Findings’ column was subdivided into ‘BOC (Quantitative)’, ‘BOC (Qualitative)’, ‘Oncology (Quantitative)’ and ‘Oncology (Qualitative)’ for easy comparison.

Incidence and mortality data from the Global Cancer Observatory 2022 and the Global Burden of Disease (GBD) 2021 studies were extracted directly from the datasets and can be found in Appendices A and B respectively.

### Data analysis/synthesis

Key information from each paper was identified. Articles discussing similar outcomes were grouped together. Due to the quantitative heterogeneity of outcome reporting between the included studies, a meta-analysis of results was not performed. Instead, a qualitative/narrative synthesis of findings was done to examine the data. This included performing a preliminary synthesis to describe findings which was then consolidated by exploring patterns that emerged across study results and the factors influencing these patterns. To judge the robustness of the synthesis, data sources and methodologies were scrutinised.

### Risk of bias assessment

Risk of bias (ROB) was not used to exclude any papers. However, ROB assessments were conducted for academic literature papers. The Newcastle-Ottawa Scale (NOS) was amended to appraise cross-sectional and retrospective epidemiological studies [27]. For qualitative studies and narrative reviews, the Critical Appraisal Skills Programme (CASP) tool was used [28], while case series were scored using the Joanna Briggs Institute (JBI) checklist [29].

## Results

**Fig. 1:**
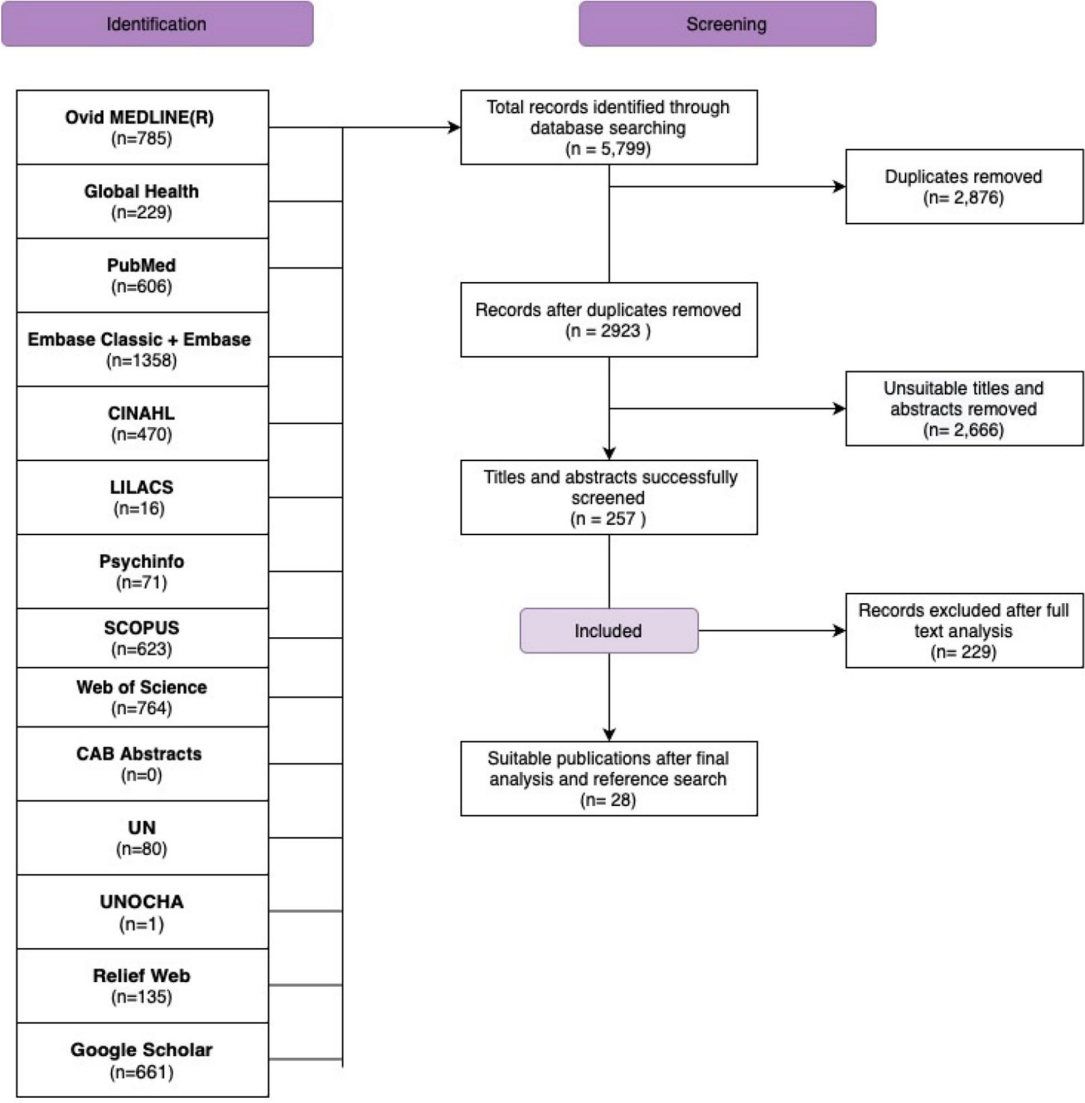
A diagram showing the study selection process to identify eligible studies

A search of the literature yielded 5,799 results. After removing duplicates and screening through abstracts and full texts for study eligibility, 23 academic papers were identified for inclusion [15, 18, 23, 30–49]. The reasons for exclusion after full-text screening were: outcomes not of interest (*n* = 129), ineligible study setting (*n* = 44), ineligible study period (*n* = 13) and ineligible study design (*n* = 37). A search through grey literature found five papers [50–54], bringing the total number of included papers to 28. Figure 1 summarises the selection process.

### Study characteristics

Of the 23 academic papers, eight were of a retrospective epidemiological study design. These typically reported incidence, prevalence, and mortality (IPM) of cancers at a national population level for Syria compared against other Asian or Eastern Mediterranean (EM) countries. Of the remaining nine academic papers, five studies covered BOC or oncology services in Damascus hospitals, one study focused on oncology services in Aleppo, and three studies surveyed oncology professionals from across Syria. These nine studies were of the following study designs: case series (*N* = 5), cross-sectional (*N* = 3) and narrative review (*N* = 1).

The five included grey literature papers were published by WHO and its affiliates as well as a report by the Syrian American Medical Society (SAMS) co-published with Relief International (RI) [50]. The included studies gathered information by extracting data from the Ministry of Health or international studies such as GLOBOCAN and through surveys or interviews with Syrian health workers or patients across Syria.

### Burden of cancer

#### Cancers at a national level

Of the seven academic studies measuring BOC for Syria at the national population level, the majority were retrospective epidemiological studies [30–36]. Key findings of studies reporting BOC are summarised in Table 3.

**Table 3:** An overview of the key findings from papers reporting on the Syrian burden of cancer

	First Author & Year	Study Design	Study Period	Study Setting & Population	Outcome Measure	Annual Incidence Rate			Annual Mortality Rate				Other Key Findings		
						Crude*	ASR*	Cum. Risk**	Crude*	ASR*		Cum. Risk**			
**National vs International Comparison**	Chattopadhyay [30]	Ecological	2012	23 EM countries	IPM lip & oral cancer	1.4	2	0.22	0.6	0.8		0.09			
	Sadeghi [31]	Ecological	2012	47 Asian countries	IPM testicular cancer	1.3	1.4		0.6	0.8			•5th highest ASR mortality rate in Asia		
	Ghoncheh [32]	Ecological	2012	47 Asian countries	IPM colorectal cancer	10.8	16.2		7.1	10.8					
	Kulhanova [33]	Ecological	2012	22 EM countries	Most prevalent cancer types in each country								Most prevalent cancer types in Syrian population:		
													•Men - 1st) Lung 2nd) Colorectal 3rd) Bladder		
													•Women - 1st) Breast 2nd) Colorectal 3rd) Leukaemia		
					IPM breast cancer		52.5			21.5					
	Fitzmaurice [34]	Ecological	2005-2015	22 EM countries	IPM all cancers		103			64					
	Goodarzi [35]	Ecological	2018	48 Asian countries	IPM breast cancer		67.3			26.9					
	Kulhanova [36]	Ecological	2012	22 EM countries	Percentage reduction in all cancers if key risk factors were eliminated								•Men - 44.2% of cancers attributable to avoidable risk factors		
													•Women - 26.4% of cancers attributable to avoidable risk factors		
													•Smoking, infections, and diet amongst biggest risk factors		
**Subnational**	Al Hasan [37]	Case Series	2012-2016	37 paediatric retinoblastoma patients at Almouassat University Hospital, Damascus	Patient demographics, biomarkers, symptoms & outcomes								•48.7% diagnosed <1yr old, 16.2% at 1-2yrs old, 24.3% at 2-3yrs old, 10.8% at >3yrs old		
													•73% diagnosed at stages I/II, 27% diagnosed at stages III/IV		
													•Findings similar to international studies		
	Kakaje [38]	Case Series	2017-2018	203 paediatric ALL patients at Children’s University Hospital of Damascus University	Patient demographics, biomarkers, symptoms & outcomes								•Peak age of 5-9 years, slightly older than mean age in other international studies		
													•Over 50% of parents had low educational level		
													•High T-cell ALL, L2 & high-risk prevalence could reflect underlying factors and poor survival rate		
	Atassi [47]	Retrospective analysis	Jan - Dec 2020	Al Rai pathology laboratory, Northern Aleppo	Total number of cancers diagnosed	379							•51.9% of cancers were diagnosed in specimens from female patients		
					Breast Cancer	80							•5% of cancers were diagnosed in specimens from patients aged 17 years or younger		
					Lung Cancer	10							•Breast cancer was the most common among female specimens, accounting fro 38.3% of cancers		
					Colorectal	29							•bladder cancer was the most common among male specimens, accountign for 15.7%		
					Prostate	15							•34 breast cancer cases were diagnosed after mastectomy of which 22 specimens were at least stage III		
					Bladder	33							•16 of 33 bladder cancer specimens were high grade, of which 8 invaded surrounding muscle		
					Corpus Uteri	18							•Compared to GLOBCAN data, this study showed lower breast, lung, colorectal and prostate cancer incidence and higher bladder, uterine and cervical cancer incidence		
					Cervix Uteri	5							•The greatest difference between GLOBCAN and this data set was in lung cancer incidence (9.4 vs 2.5%), which may be due to discrepancies in availability of diagnostic equipment		
					Skin	48							•The higher rate of bladder cancer may be related to greater exposure to carcinogens in Northwest Syria due to the greater number of attacks in the area compared to other parts of Syria		
					Lymph node	35									
					Other cancers	129									
	Harfouch [48]	Retrospective analysis	2014-2018	Patients diagnosed with colorectal cancer (CRC) in Tishreen University Hospital between 2014-2018	CRC cases as a percentage of all tumours diagnosed in the hospital per year								• The highest number of CRC cases was 236 in 2018 vs the highest percentage compared to overall tumours was in 2016		
					2014	7.35%							•The number of registered CRC cases in Latakia was higher than other areas in Syria, likely due to better access to diagnostics at Tishreen University Hospital		
					2015	8.38%							•Annual incidence of 8.07% in 2018 is lower than the global incidence of 10.2% in 2018		
					2016	8.82%									
					2017	8.65%									
					2018	8.07%									
					Total	8.20%									
	Manachi [23]	Book chapter	2020	Cases from Al Bairouni hospital-based registry	Breast	24.36%							•Al Bairouni hospital sees over 60% of cancer patients in Syria, it is the only treatment centre for thyroid cancer		
					Head and neck	22.00%							•~9000 new cancer cases in the hospital in 2020, of which 56% were female		
					Lung	9.75%							•The first national screening programme for breast cancer was in October 2019 and used mamograms and guided biopsy for women between 35-70 years		
					Colorectal	7.42%							•A national cervical screening programme was started in 2021		
					Thyroid	8.31%							•The main public diagnostic cancer lab is at the Al Bairouni University Hospital		
					Cervix and uterus	4.17%							•The WHO found a 30 day waiting list for radiotherapy, 15 day for surgery and 8 day for systemic therapies in Syria in 2016		
					Bladder	3.32%									
					Prostate	2.59%									
					Testicular	0.95%									
	Baddour [49]	Retrospective analysis	2019-2020	Patients undergoing radiotherapy treatment at Tishreen University hospital between 2019-2020	Breast	298							•30% of patients were from Aleppo, 24% Lattakia, 20% Tartous, 10% Hama, 8% Homs, 3% Idlib, 2% Damascus		
					Lung	69									
					Larynx	67							•24.6% of patients were current smokers and 8.8% were previous smokers		
					Brain	57							•65.2% of lung cancer, 70.2% of laryngeal cancer and 57.9% of bladder cancer patients were smokers		
					Endometrial	49									
					Rectal	31									
					Prostate	30									
					Bladder	19									
					Hodgkins lymphoma	18									
					Lip	12									
					Pharynx	11									
					Skin	11									
					Other	121									

*ALL *Acute Lymphoblastic Leukaemia, *ASR *Age-Standardised Rate, *EM *Eastern Mediterranean, *IPM *Incidence, Prevalence, Mortality, *WHO *World Health Organisation

*per 100,000 people

**% cumulative risk of event before 75yrs old

Most studies reported data from GLOBOCAN [35–42]. According to GLOBOCAN 2022 estimates, the overall age-standardised rate (ASR) incidence of cancer was at 135.7 per 100,000, with overall ASR mortality at 89.6 per 100,000 in 2022 (see Appendix A). Incidence and mortality saw a 31.7% increase and 40% increase, respectively, when compared to 2015 estimates from the GBD study [34]. The large differences in incidence and mortality rates may have been due to the lack of high-quality data and discrepancies in collection methods. Due to the absence of a national cancer registry in Syria from 2011 onwards, GLOBOCAN IPM estimates were calculated by extrapolating mortality: incidence data from neighbouring countries [87]. The GBD study, meanwhile, used mortality rates from vital registration data to then estimate incidence. There is also variation between both methods in the coding for different cancers which can impact estimates [88]. These variations in data collection and sources could result in potential sampling and measurement biases making the data from these studies less reliable [89]. This also impacts longitudinal analysis of cancer burden in Syria as comparisons between the two studies may not be valid due to the significant variation in research methods.

#### Breast cancer

Two papers addressed the burden of breast cancer at a national level. Breast cancer was the most common cancer for women in Syria in the years 2012, 2018 and 2022 [33, 35]. According to GLOBOCAN estimates, ASR incidence and mortality rose by 28.2% and 25.1%, respectively, between 2012–2018 [33, 35]. From 2018 to 2022, incidence and mortality fell by 31.4% and 22.3% respectively ([35], Appendix A).

#### Other cancers

Three papers reported the IPM of other cancers in 2012 [30–32]. When compared to GLOBOCAN 2022 data, colorectal cancer ASR of incidence and mortality decreased by 32.7% and 33.3%, respectively [32]. The ASR of incidence for testicular cancer rose by 42.9% between 2012 and 2022, although the ASR of mortality dropped by 37.5% [31]. In the same period, the ASR of incidence of oral cancer dropped by 35% while the ASR of mortality rose by 12.5% [30].

#### Cancers at a subnational level

Six papers examined BOC at a subnational level [23, 37, 38, 47–49]. Al Hasan et al. looked at the demographics of 37 paediatric retinoblastoma patients at Almouassat University Hospital [37]. Sex ratios (1.6 M/F), proportion of hereditary cases (24.3%) and proportion of cases diagnosed at stages III/IV (27%) were all consistent with similar studies from Mexico and Saudi Arabia [55, 56]. Kakaje et al. reported on the demographics of 203 paediatric acute lymphoblastic leukaemia patients in Damascus [38]. Although age and sex ratios of patients were similar to those in comparable populations, there was a higher proportion of high-risk patients (48.4%) and their prognosis was typically poorer [57, 58].

Atassi et al. retrospectively analysed histopathology reports for all cancer specimens from the Al Rai pathology laboratory serving NWS between January and December 2020 [47]. The most common cancer in women was breast cancer (38.3%) and in men was bladder cancer (15.7%). Of the 33 bladder cancer specimens, 48.5% were high grade and 24.2% were locally invasive. Of the 34 breast cancer specimens 64.7% were stage III or above, which is similar to findings from Nahhat et al.’s study that reviewed medical notes for breast cancer patients at Al Bairouni University Hospital in Damascus, between January 2019 and May 2022 and found that 61% of patients were stage III or above at diagnosis [18]. Manachi et al. reviewed cancer cases from the Al Bairouni University Hospital based registry in 2020. They found that breast (24.4%), head and neck (22%) and lung cancers (9.8%) were the top three cancers recorded at the hospital in that year [23].

Baddour and Al-Mahmoud [49] reviewed cases of patients undergoing radiotherapy treatment at Tishreen University Hospital between 2019 and 2020. They found the most common cancer treated was breast cancer and that the greatest proportion of patients treated hailed from Aleppo (30%). Of note, 33.4% of patients were current or previous smokers and smokers made up 65% of lung cancer, 70% of laryngeal cancer and 58% of bladder cancer patients [49]. Harfouch et al. retrospectively analysed medical notes of patients diagnosed with colorectal cancer in Tishreen University Hospital in Lattakia between 2014 and 2018. They noted an increase in the number of colorectal cancer cases as a percentage of all tumours of 0.72% from 2014 to 2018 [48]. However, the number of colorectal cases as a percentage of all cancer cases in the hospital in 2018 was 8.07%, which is lower than the global comparison of 10.2%.

### Oncology services

Fifteen papers discussed the quality or availability of oncology care, four of which were WHO reports [15, 18, 39–46, 50–54]. These examined outcomes falling under five main themes: hospital and clinic availability (*n* = 6) [15, 41, 46, 50, 51, 54], adequacy of staffing (*n* = 6) [15, 41, 42, 50, 51, 53], resource and treatment availability (*n* = 13) [15, 39–44, 46, 50–54], affordability of care (*n* = 6) [15, 40, 41, 50–52], and availability of screening and palliative care (*n* = 8) [15, 18, 41, 45, 51–53]. Key findings from these studies are outlined in Table 4.

**Table 4:** An overview of key findings from papers reporting on Syrian oncology services

	First Author & Year	Study Design	Study Period	Study Setting & Population	Outcome Measure	Summary of Key Findings	
						Provision	Challenges
**Academic Literature**	Salamoon (2013)	Case Series	2012	236 sarcoma patients at Al-Bairouni Hospital, Damascus	Response and survival rate to cisplatin instead of anthracycline in chemotherapy regimen		• Shortage of anthracyclines for chemotherapy in Damascus
							• Cisplatin used as substitute; metastatic tumours responded well
	Rajeh [40]^a^	Case Series	2012-2013	17 stem cell transplantations in private sector, Damascus	Success, side effects and survival rate of stem cell transplantation		• Stem cell transplantation facilities limited across Syria
							• Major medical shortages due to travel restrictions and embargoes
							• Syrians having to 'pay out of pocket', health insurance also underdeveloped
	Sahloul [41]	Cross-Sectional	2015	General practising physicians and oncologists working across Syria	Staffing, availability, quality, and cost of treatment		• Lack of specialised oncologists, anti-cancer drugs, radiotherapy, screening programmes and follow-up services
							• Telemedicine and patient education are important solutions in rebuilding the healthcare system
	Patenaude [42]^a^	Case Series	2011-2017	Department of Paediatric Oncology at Al Bairouni Hospital, Damascus	Psychological impact of war on paediatric cancer treatment		• Lack of medication, facilities, transport as well as high costs can cause heavy emotional burden to patients and parents
							• Exposure to war and worry for safety of loved ones affected health workers' attention, availability, and energy
	Alhames [15]	Narrative Review	2012-2020	Healthcare services in Aleppo	Quality and availability of cancer care in Syria, particularly Aleppo	• Aleppo slowly rebuilding since 2016, but still inadequate facilities	• Lack of specialised oncologists, anti-cancer drugs, radiotherapy, follow-ups
							• Healthcare unaffordable for many
	Benbrahim (2020)	Cross-Sectional	2020	Senior oncologists from 13 EM countries, including Syria	Response of oncology services to COVID-19 pandemic	Safety measures are in place in Syria, e.g.: screening patients for COVID, masks, social distancing, disinfecting surfaces	
						• Action plans are in place to treat COVID-positive patients	
	Abdul-Sater [44]	Qualitative interviews	2019	Key informants from Lebanon, Syria, and Iraq. 3 from Syria	Challenges to cancer research		• Drug and equipment shortages (due to sanctions) make clinical trials difficult
							• Syrian war has caused increased poverty and decreased cancer healthcare provision
							• Lack of radiotherapy facilities
	Bruni [45]	Review and synthetic analysis	2019	202 countries including Syria, women aged 35-49	Status of cervical screening programmes and their coverage on a country level	• 5% of target population undertook cervical screening between 2014-19 (77,844 women)	• A minimum of 1,124,475 women need to undergo cervical screening between 2019-24 to achieve the WHO 70% screening target
	Nahhat [18]	Retrospective analysis	Jan 2019 - May 2022	Medical notes for breast cancer patients treated at Albairouni University Hospital	Method of screening and diagnosis, stage at diagnosis	• 65% of patients were diagnosed by surgical procedure (lumpectomy/mastectomy) rather than biopsy	• 95% of patients had not enrolled in screening programmes before diagnosis
							• 42% were stage III at diagnosis, 31% stage II, 19% stage IV
							• Metastatic disease was more commonly found in patients found from Homs and Hama than other areas
	Hanafi [46]	Cross-sectional retrospective cohort study	Aug-Sep 2019	519 breast cancer patients at Al-Bairouni Hospital, Damascus	stage of cancer at diagnosis, symptoms at presentation, effect of accessibility of healthcare on diagnosis and treatment		• Most common cause for delay in presentation was lack of knowledge (26.4%), followed by problems related to the healthcare system (19.46%) • Lack of accessible healthcare was the only variable that significantly impacted total delay in management • ~37% of patients reported a delay of >3 months for management, mostly related to either lack f knowledge or inaccessible healthcare• 20% of patients report lack of access to healthcare due to armed conflict• 55% of patients presented with advanced cancer
**Grey Literature**	WHO (2016)	Report	2016	Head of hospital, oncologists, and oncology workers at 8 hospitals under government control in Syria	Quality and availability of cancer care		• Setting up national cancer registry
							• No national cancer control plan
							• Limited staffing, lack of training programs for staff
							• Shortage of cancer drugs, lack of radiotherapy in Aleppo & Suwayda
							• Lack of community-based palliative care
	WHO (2017)	Report	2017	Syria	Quality and availability of cancer care	• Cancer centres, pathology, surgery, chemo, radiotherapy all available in public sector, but no mention of level of coverage	• Lack of national action plan/strategy for cancer, vertical programmes for cancer, public sector palliative care
							• Limited screening available for breast and cervical cancer
	WHO (2020)	Report	2020	Syria	Availability of cancer care	• Breast and cervical cancer screening available	
						• 3 public cancer centres per 10,000 cancer patients	
						• 12.9 medical physicists (focusing on cancer) per 10,000 cancer patients	
	WHO HeRAMS (2020)	Report	2020	Syria	Availability of cancer care in public hospitals	• 48% of public hospitals fully functioning, 28% partially functioning, 24% non-functioning	• Limited anti-cancer drugs
						• 29% of people serviced by cancer treatment services	
						• Cancer consultations rose by 12.2% from 2019 to 2020	
						• Majority of consultations and treatment occurs in Damascus	
	SAMS and RI (2023)	Advocacy Brief	Oct 2021 - March 2023	Northwest Syria	Quality and availability of cancer care	• There are an estimated 2-3,000 new cancer diagnoses made in NWS each year	• The majority of healthcare in NWS is currently supported by NGOs and as cancer care is considered tertiary healthcare it is not funded by donors so it is very poorly funded
						• Most hospitals in NWS have the capacity to perform resective and exploratory surgeries but for advanced treatments such as radiotherapy patients must be referred to hospitals outside of Syria	• In a survey by RI in Idlib and Aleppo in April 2022, 65% of women did not know where to go for mammogram screening
						• Ultrasound and radiography is provided in five health facilities in Aleppo and nine in Idlib governorates	• The waiting time between referral and treatment in Turkey is between one and six months
						• In 2022 there were seven CT scanners, one MRI machine and four mammograms in NWS	• General laboratory testing during treatment cycles is not provided by NGOs
						• Free pathology testing is avilable at 1 hospital in NWS	• Medical referrals to Turkey has decreased yearly since the Covid-19 pandemic
						• Chemo- and hormonal therapy are provided at 3 centres in NWS	• Referral for radiotherapy in Turkey has stopped since the earthquake in February 2023

*EM *Eastern Mediterranean, *HeRAMS *Health Resources and Services Availability Monitoring System, *WHO *World Health Organisation, *SAMS *Syrian American Medical Society, *RI *Relief International, *NWS *Northwest Syria, *CT *Computed Tomography, *MRI *Magnetic Resonance Imaging

^a^abstract only

#### Distribution of services

**Fig. 2:**
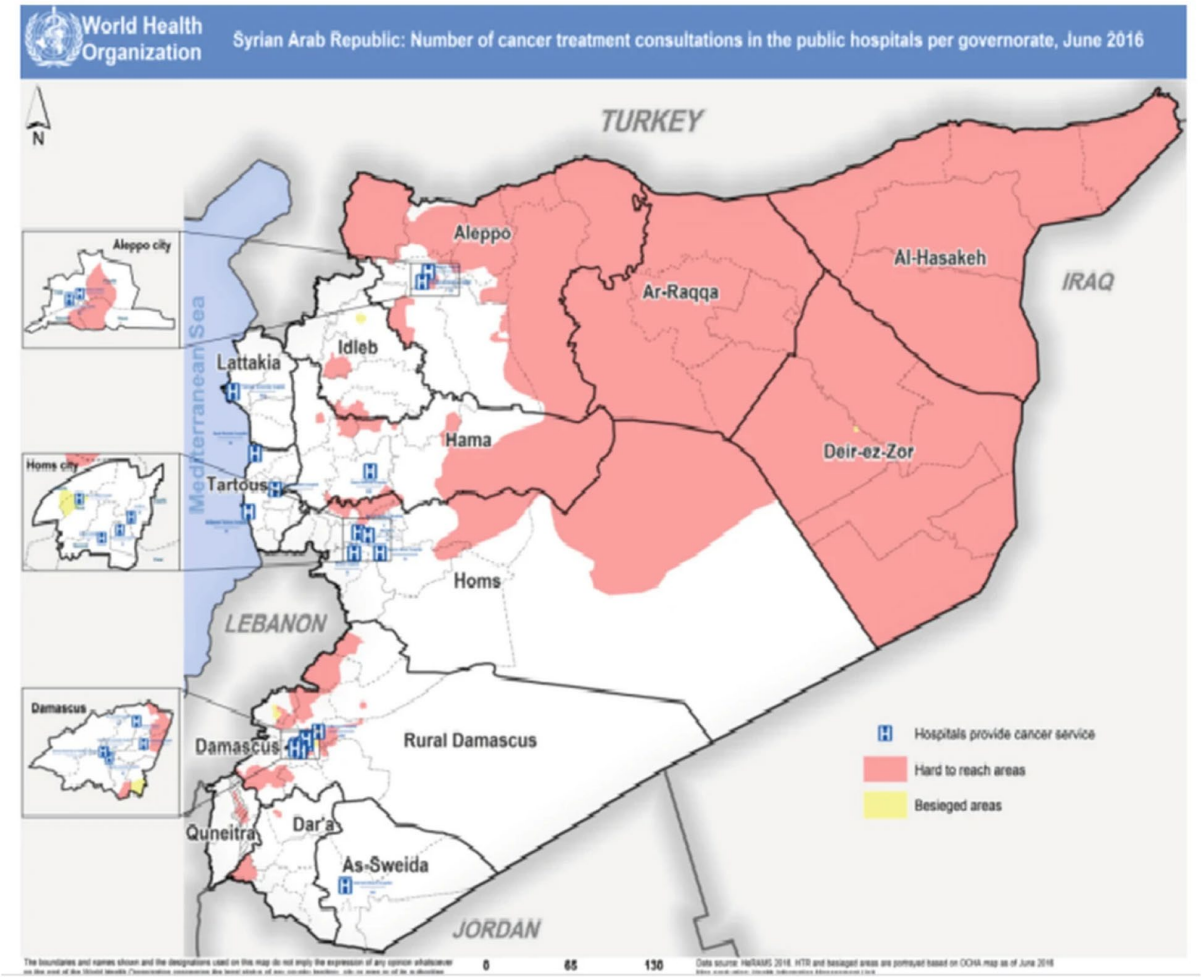
This map from WHO shows the main cancer centres across Syria. It is from 2016 but the main cancer centres in areas under government control remain the same; there are still no cancer centres in northeast Syria and only one cancer centre (Idlib Oncology Centre) in northwest Syria [23]

The WHO Health Resources and Services Availability Monitoring System (HeRAMS) report in 2020 found the availability of medicines for cancer and the availability of cancer treatment services across Syrian public hospitals to be 19% and 29% respectively [54]. Oncology services were largely concentrated around government controlled Damascus and Rural Damascus, where 89.6% of cancer consultations took place. Like many countries in the region and internationally, cancer services tend to be concentrated in major urban centres, particularly Damascus, Aleppo, Homs, Lattakia and Tartous. Figure 2 shows the location of the different cancer centres throughout Syria. However, the centres did not provide the same range or level of services with Damascus and Aleppo providing the most advanced cancer care. Since the conflict, some of the decentralisation of services has been reversed; for example, Al-Bayrouni centre in Damascus is estimated to handle more than 60% of total oncology care [23]. Faris et al. ‘s WHO report surveying eight government-controlled hospitals in 2016 found Al-Bairouni Hospital in rural Damascus to provide the most cancer consultations, followed by hospitals in Lattakia and Damascus respectively [51]. Manachi et al. report that Al Bairouni hospital manages 60% of cancer patients in Syria and that it is the only thyroid cancer treatment centre in the country [23]. Nahhat et al. in their review of medical notes of 2,367 breast cancer patients in Al-Bairouni Hospital found that patients from Homs and Hama were more likely to present with metastatic disease than patients from other areas of Syria [18].

The lack of radiotherapy facilities in Syria was a recurring theme across the studies, with absence of facilities in Aleppo governorate forcing patients to travel to Damascus and Lattakia, or abandon treatment entirely [15, 41]. The 2023 report by SAMS and RI highlights the difficulties of providing cancer care in NWS which is mainly non-government controlled and where the majority of healthcare is provided by non-governmental organisations (NGOs) and funded by international donors. They report that most hospitals in the area provide diagnostic and resective surgeries for cancer but there are only three centres that provide basic chemotherapy regimens and radiotherapy treatment is not available in NWS. Patients who require radiotherapy are referred to treatment centres in Turkey with referrals taking between one and six months before treatment is started [50]. SAMS established an oncology centre in Idlib city in 2018 which is the only specialised centre providing oncology care for the population of non-government controlled NWS [47].

#### Adequacy of staffing

A paper by Sahloul et al., surveying 35 oncologists across Syria in 2015, found that only Damascus met the National Cancer Institute’s guidelines for staffing levels [41]. Staffing between cities was highly variable, with over 20 specialised oncologists (subspecialty not known) found in government-controlled Damascus and just 6 located in the similarly populated city of Aleppo. They were all in government-controlled west Aleppo, making it very difficult for cancer patients in rebel-held east Aleppo to seek treatment. Faris et al. found the lack of specialised oncologists and nurses to be a ‘root challenge’ [51]. The average numbers of medical oncologists and surgeons conducting cancer operations were 5 and 9 respectively, although these numbers may have been skewed upwards by the inclusion of major cancer centres. The WHO Cancer Country Profile 2020 found 12.9 medical physicists to be available per 10,000 cancer patients in Syria, slightly higher than the global median of 12.0 and in line with averages for the EM region [53]; however given the high proportion of healthcare workers who have left Syria since 2011, this figure may have significantly overestimated staffing numbers. Patenaude et al. found that worry for the safety of family and friends during the conflict profoundly affected the psychological state of healthcare workers [42]. The SAMS and RI 2023 advocacy brief reports that there are only 35 oncologists currently in Syria and they cite inadequate staffing as one of the main factors leading to poor cancer care for patients [50].

#### Resource availability

The lack of cancer medicines was addressed in seven papers [15, 39–41, 44, 51, 54]. Essential cancer medicines were sometimes available in major cities such as Damascus and Aleppo, but shortages did occur, typically once or twice per month [51] and often caused delays in treatment for up to several months [15]. A 2013 case series reported drug shortages in Damascus forcing regimens to be modified [39]. Furthermore, availability of more advanced cancer medicines such as targeted therapy was limited across all of Syria including Damascus [15, 41]. Unreliable supply chains, poor and unsafe transport networks [44] and economic sanctions [15] were reported to be responsible for these shortages, with one study reporting that transporting medications into besieged areas was especially difficult [41]. Benbrahim et al.’s study on national responses to the COVID-19 pandemic found that there were guidelines for oncology services in Syria to protect against infection spread, including use of personal protective equipment; however, there was no mention of the implementation of these guidelines [43]. SAMS and RI report that in 2022, there were seven computed tomography (CT) scanners, one magnetic resonance imaging (MRI) machine and four mammograms to serve the entire population of NWS which is mainly non-government controlled [50]. They also report that in NWS, there are five health centres in Aleppo and nine health centres in Idlib that provide ultrasound radiography diagnostic imaging for the population. Hanafi et al. in their cross-sectional study of 519 breast cancer patients at the government-controlled Al-Bairouni Hospital in August to September 2019 found that lack of accessible healthcare was the only factor to have a significant impact on total delay in management. Of their patient cohort, 55% presented with advanced cancer and 20% reported reduced healthcare access due to armed conflict [46].

#### Affordability of care

Three studies cited the affordability of oncology care as a major challenge faced by most Syrian cancer patients during the years of conflict. Patients were often forced to pay out-of-pocket due to near absence of health insurance coverage [15, 40, 41]. In their survey of government-controlled hospitals, Faris et al. found that 15% of total cancer care costs were paid out-of-pocket by patients and just 3% were covered by health insurance [51]. Another study in 2016 found that costs of cancer care ranged between $100-$1000 monthly, which was reported to be too expensive for the average Syrian. This was a particular issue in rebel-held areas, as patients could not be referred to the two treatment centres in Damascus where costs were covered by the government [41]. The WHO Country Capacity Survey (CCS) in 2017 did mention key services such as radiotherapy and subsidised chemotherapy being available in public hospitals, but there was no reference to the level of coverage [52]. In non-government-controlled NWS, according to the report by SAMS and RI, where healthcare provision is funded by external donors, cancer care is considered tertiary level healthcare and as such is not funded by these donors and NGOs are required to fundraise independently for these provisions [50]. Therefore, the availability of free of charge diagnostic and treatment services for cancer is very limited for a population where 90% live below the poverty line [59].

#### Screening

Several papers mentioned the presence of cervical (pap smear) and opportunistic breast (mammogram) cancer screening programmes [15, 18, 41, 45, 50–53]. One paper reports that as of 2015, these were only available in Damascus [41]. In contrast, Manachi et al. [23] in their book chapter report that the first breast cancer screening programme in Syria was started in 2019 and that a national cervical screening programme was initiated in 2021 [23]. In Nahhat et al.’s study of breast cancer patients, they found that 95% had not enrolled in a breast screening programme before diagnosis and 61% of patients had stage III or more advanced breast cancer at diagnosis [18]. In Bruni et al.’s study looking at the status of cervical screening programmes in 202 countries, they found that in Syria 5% of the target population undertook cervical screening between 2014 and 2019 [45].

#### Palliative care

Three studies cited a lack of palliative care across Syria, both in primary care and the community [15, 51, 52]. Although Faris et al. found that 88% of inpatient settings had provided palliative care, availability was lower in outpatient (75%) and community-based (63%) settings. They reported that inadequate funding (88%) and lack of staffing (88%) were the main barriers to services. The scarcity of resources suggests that palliative care was of low priority to the Syrian Ministry of Health [51].

### Risk of bias assessment

Fifteen papers were appraised using NOS. These are shown in appendix C. Eight studies scored 8/10. Data ascertainment and analysis were robust as was sampling. However, all but one failed to provide adequate information about non-respondents. Benbrahim et al. [43] scored particularly poorly (3/10) due to poor sampling and recruitment and Manachi et al. (2/10) due to very limited information provided on methodology [23].

Five cases-series were appraised using the JBI checklist as shown in Appendix D. Most papers (*n* = 4) used valid methods to identify illness and monitor clinical information of participants. However, many papers (*n* = 4) lacked sufficient information about data completeness and participant demographics. Two studies performed especially poorly, although this was expected as only the abstracts of these papers were available [40, 42].

The two papers being assessed by the CASP tool showed similar weaknesses. Both failed to provide sufficient information to judge the appropriateness of their respective methodologies. This can be seen in Appendix E.

## Discussion

This systematic review provides an insight into the BOC and the state of oncology healthcare services in Syria throughout the course of the conflict, starting in 2011. Although 28 papers were identified, broadly, there was a dearth of available data of sufficient quality and little in-depth exploration of cancer services, or how they have been affected by the conflict. Of note, there was also limited examination of BOC in different areas of geopolitical control, as well as of the state of oncology services in these micro-contexts.

### Burden of cancer

When analysing the BOC in Syria, the heterogeneity of outcome measures and lack of comprehensive data makes it difficult to draw definitive conclusions. Without data on IPM of cancers for each year of the conflict, it is difficult to thoroughly analyse longitudinal changes in cancer rates. Moreover, the absence of a national cancer registry since 2011 means that all national data sources, including GBD and GLOBOCAN studies, rely on poor quality data and extrapolating IPM from neighbouring countries using varying methods, making them inaccurate [15]. Moreover, the fragmentation of Syria’s healthcare system during the conflict has now heightened the need for an authoritative cancer surveillance system at both local and national levels. As provision of oncology services vary greatly between regions, health outcomes for cancer patients are likely to be affected [15, 41]. Yet the lack of regional data makes it difficult to assess the true extent of this impact. To guide policymakers in the rebuilding of regional healthcare systems, more comprehensive data and prospective studies are required. This is especially needed in areas such as the northeast and northwest of Syria, which have been hit hardest by the conflict and for which there is very little information [67].

With an ASR of incidence of 135.7 per 100,000 in 2022, Syria’s incidence rate was far higher than the EMRO (Eastern Mediterranean Regional Office) regional average of 127.2 [60], according to GLOBOCAN data. Studies looking at the demographics of cancer patients in Damascus hospitals [37, 38] found similarities in many characteristics such as sex and age compared to international studies [55, 57, 61], but also observed a greater proportion of high-risk patients and worse prognosis [57, 58]. This may be related to delayed presentation due to limited access to specialist services, limited financial capacity to attend clinics or fears surrounding the impact of these diagnoses on working potential. Additionally, it could be due to patient concerns regarding the price of treatments or poor management of comorbidities due to a myriad of reasons such as limited access to healthcare, insufficient medication supplies and limited financial capacity of patients.

The only study to review data for patients with cancer in NWS found, similar to other studies in Syria, that breast cancer was the most common cancer in women and bladder cancer in men [47]. They also found that 64.7% of breast cancer specimens were stage III or above at diagnosis, similar to the findings from Nahhat et al.’s study in a Damascus hospital that found 61% of patients presented with stage III or above breast cancer [18, 47]. This is a concerning finding when considering the higher mortality and morbidity associated with later stages of cancer, particularly as it is replicated in both government controlled and non-government controlled areas of Syria. As a comparison, researchers from the IARC found that for breast cancer patients diagnosed after 2015, the global estimate of patients diagnosed with stage III or above cancer was between 3 and 12% [62] and in England in 2021, 12.7% of breast cancer patients were diagnosed at stage III or above [19].

The overall rise in cancer incidence and mortality may be indicative of worsening health outcomes for Syrians, with the conflict causing declines in living standards and greater exposure to health hazards, particularly amongst internally displaced people. This may include severe malnutrition (particularly in view of the cuts to the World Food Program) [63], the lack of adequate shelters, the burning of harmful plastics [64] or stress-related smoking rates [65]. Such factors have been highlighted by Kakage et al. who found (among other factors) that conflict may be a contributor to higher mortality rates among leukaemia patients [38]. Atassi et al. found that the rates of bladder cancer in their study in NWS in 2020 were higher than GLOBOCAN estimates for Syria in the same period. They hypothesise that this may be due to greater carcinogen exposure of the population in NWS due to the greater number of attacks on the area as compared to other parts of Syria throughout the conflict [47]. Similar problems were seen amongst Syrian refugees in Turkey, where poor hygiene and living standards, compounded with poor accessibility to healthcare, resulted in late diagnosis and interrupted treatment [66]. However, the BOC among refugees remains unknown, with poor access to diagnostics leading to underdiagnosis [24].

The findings of this review that the BOC in Syria is poorly defined is not unexpected, given that there is evidence of similar challenges in other protracted conflict settings, where healthcare access and cancer surveillance is limited. In neighbouring Iraq for example, cancer surveillance was hampered by the fragmentation of registry systems during military conflict [68]. In Lebanon, meanwhile, civil war caused the closure of diagnostic facilities, making data ascertainment difficult [68]. Challenges in monitoring BOC can also last beyond the end of conflicts, with difficulties found in re-establishing screening and registry systems across various African post-conflict settings [69, 70]: a cautionary tale for Syria in its current state of frozen conflict and possible post-conflict transition.

### Provision of oncology services

With regards to oncology services during Syria’s conflict, some themes recurred across the literature. Shortages in staffing [15, 41, 51], chemotherapeutic drugs [15, 39–41, 44, 51, 54], and radiotherapy [15, 41] were found to be endemic during the conflict. But there is significant study bias with little to no comment or examination of surgical services, palliative care, childhood cancers, haemato-oncology or other types of site-specific cancers. Moreover, most papers focused on the availability of very select types of oncology supplies and services. While all types of cancer services associated were reported to be lacking across the entire country often due to inadequate funding and staffing [15, 41, 51–53], available oncology services are mainly concentrated around government-controlled areas including Damascus and Lattakia [15, 41]. The situation is particularly dire in NWS where oncology care is seen as tertiary level healthcare and as such is not funded by external donors. Consequently, the provision of oncology care is incredibly limited, with most patients requiring referral to outside of Syria for any advanced oncology treatment such as radiotherapy [50, 71].

For oncology services, most of the data were collected at a national level or from Damascus and Aleppo cities. Only four studies collected data from other regions in Syria [41, 50, 51, 54]. This makes it difficult to gain an accurate understanding of the state of cancer care across the entire country, and in different areas of political control. Availability of cancer services varied across time and geographical regions. In particular, there was a large gap in services between Syria’s two largest cities, Damascus, and Aleppo. Damascus, historically a government stronghold, was already the location of numerous cancer specialist facilities, including Al-Bairouni hospital [23, 50]. Apart from certain areas, Damascus was largely secure from the most intense fighting during the conflict, and government forces were able to consolidate control over most of the region by 2014 [72]. Aleppo’s healthcare in comparison, was historically not as well funded or staffed [73, 74]. The governorate also endured some of the most intense fighting of the conflict between 2012 and 2016, and conflict continued in the region until 2019 [75]. As the city endured a 4-year siege as well as targeted airstrikes on healthcare facilities, Aleppo was left with no functioning hospitals and very few healthcare workers by 2016 [15]. This almost complete destruction of facilities likely stunted the reconstruction of healthcare services. Moreover, as the city became divided into government-held west Aleppo and rebel-held east Aleppo, we can also see a divide in the availability of oncology services, as demonstrated in Sahloul et al.‘s study [41]. The loss of healthcare workers and the difficulty of bringing medical supplies into besieged east Aleppo meant that oncology services in east Aleppo were absent. Although west Aleppo has been able to rebuild some services slowly, this is still far less comprehensive as compared to the traditional government strongholds of Damascus and Lattakia [41].

The lack of multimodal cancer services in areas like Aleppo governorate force many Syrians to take long journeys into Damascus or Lattakia to receive essential treatments such as radiotherapy or to be sent out of the country to health centres in neighbouring countries like Turkey. These journeys are often unsafe for Syrians living in devastated parts of the country [15, 41]. The cost of travel in addition to treatment is of particular concern considering the rising cost of living and Syria’s economic woes. Syria’s national economy declined by 70% between 2011 and 2017, and the country witnessed soaring inflation rates [76]. Sahloul et al. found that monthly cancer care costs could reach as high as $1500, while the median Syrian monthly salary was just $150 in 2017, making treatment costs prohibitive [41]. SAMS and RI in their advocacy brief in 2023 cite several social factors that stopped women in NWS from attending breast screening including far distances to travel to clinics and limited income to pay for screening or the travel to and from [50]. The ‘financial toxicity’ for cancer patients in NWS is described by Al-Abdualla et al. who suggests a different model of humanitarian funding to support cancer care in such settings is needed [11].

Similar problems were seen in neighbouring Lebanon, where high inflation rates pushed up treatment costs and forced facilities to shut down [77, 78]. As of 2020, Lebanon actually had less public cancer centres (2.9) per 100,000 than Syria (3.0) despite not directly hosting any conflict [79]. In Syria however, most free cancer treatment was exclusive to government-controlled areas and not available to those living in opposition-held territories [15, 41]; this only entrenched the divide in oncology availability between government and opposition-held regions.

Very little information was found regarding the quality of services or on the patient experiences. Given the potential of the conflict to contribute to delays in diagnosis, research which examines this could be important in understanding patient pathways and barriers to access. As for public health measures for cancer, several papers acknowledged the presence of screening programmes in Syria which were often limited in scale and efficacy. Ostensibly breast and cervical screening programs were reported as being delivered in line with WHO recommendations, but this cannot be verified [80, 81]. services were likely opportunistic and with limited coverage. Moreover, these programmes were exclusive to Damascus, which, again, highlights the massive inequity of service distribution across Syria. Inadequate screening programmes were found in neighbouring Jordan and Lebanon as well, both of which have recently had to deal with intense economic pressure and the mass influx of Syrian refugees [52, 78, 82]. Limited palliative care provision was also reported across Syria, although a dearth of services can be seen across the entire EM region, which is suggestive of other influencing factors aside from the conflict [52]. This trend could be attributed to the low prioritisation of palliative care by policymakers amid economic and political instability in the region [51], but other factors such as cultural contexts should also be considered [83].

### Recommendations

Due to Syria’s protracted conflict and collapsed economy the challenges are greater and the approach to making improvements must be customised to its unique situation. Investment in prevention programs such as nationwide HPV vaccination, screening programs like breast screening programs, and improving public awareness of signs and symptoms as well as preventable risk factors are all effective interventions to reduce the burden of cancer. Capacity building for medical staff in oncology is essential, along with establishing a cancer registry system or incorporating oncology data collection tools within humanitarian responses. Additionally, mapping available oncology services in Syria and strategically supporting partners who provide treatment can enhance care. This support should focus on enabling diagnosis, surgeries, and provision of selected medications from the WHO Essential Medicines List, and establishing referral oncology centres in strategic locations to improve access [90].

### Limitations

There are a number of limitations to the findings in this systematic review. The heterogeneity of outcome measures and study methods prevented us from conducting a quantitative meta-analysis. Though we identified 29 papers, few were of high quality or sufficient size to offer an accurate and comprehensive account of the state of oncology services and cancer in Syria. Challenges of research in conflict zones such as limited access to data, limited diagnostics and risks surrounding identification of components of research articles complicates the publication of reliable studies and therefore limits access to high quality data. Most data were collected from Damascus and Aleppo cities, and the poor availability of regional data, particularly in the northeast and northwest of Syria, limited the ability to fully explore the BOC and oncology services in these areas. Of note, these areas had been particularly affected by the protracted conflict and continue to be [84]. Furthermore, having more comprehensive data on cancer rates in Syria would be desirable in gaining a better understanding of the true BOC. We were unable to obtain GLOBOCAN datasets for previous years despite contacting IARC for permission, as they had been removed from public access due to continuous development of estimation methods meaning that estimates from different years cannot be reliably compared [91]. Nevertheless, having the most recent version of the GLOBOCAN study allowed us a somewhat broad comparison of IPM estimates between key cancers. Despite these limitations, the findings of this review offer a useful addition to what is published about cancer in Syria during the last decade of conflict.

## Conclusion

The conflict in Syria has profoundly disrupted the country’s oncology services and exacerbated long-standing inequities in healthcare distribution across Syria. However, research output on this topic within Syria remains limited and the country lacks high quality data sources to record and track the BOC. This makes it difficult to assess the true impact the conflict has had on Syrian cancer patients. As the country continues to rebuild oncology services, there is an urgent need to conduct more research to understand how the burden has changed, particularly for vulnerable groups (women, children and socio-economically disadvantaged), how cancer services and systems have been impacted and broader questions related to the funding and re-building of national cancer control. There is an urgent need to re-establish a national cancer registry across the whole of Syria including areas outside of government control to better understand the current situation and model an operational plan for rebuilding cancer services over the next decade. Given the protracted nature of this conflict, the need to fulfil the role of the Ministry of Health in areas outside of government control, and the need for high-cost services which would not otherwise form part of a humanitarian response, policymakers should strengthen cancer services across Syria, particularly in geographical areas which are neglected.

## Supplementary Information

Supplementary Material 1.

Supplementary Material 2.

## Data Availability

All data generated or analysed during this study are included in this published article [and its supplementary information files].

## Abbreviations

ASR: Age-Standardised Rate
IPM: Incidence, Prevalence, and Mortality
BOC: Burden of Cancer
CASP: Critical Appraisal Skills Programme
JBI: Joanna Briggs Institute
GBD: Global Burden of Disease
GLOBOCAN: Global Cancer Incidence, Mortality and Prevalence
IARC: International Agency for Research on Cancer
SAMS: Syrian American Medical Society
RI: Relief International
HeRAMS: Health Resources and Services Availability Monitoring System
NGOs: Non-governmental organisations
IDPs: Internally Displaced People
EM: Eastern Mediterranean
EMRO: Eastern Mediterranean Regional Office
MeSH: Medical Subject Heading
NCD: Non-Communicable Disease
NWS: Northwest Syria
NOS: Newcastle-Ottawa Scale
PRISMA: Preferred Reporting Items for Systematic Reviews and Meta-analyses
ROB: Risk of Bias
WHO: World Health Organization
LILACS: Latin American and Caribbean Health Sciences Literature
CINAHL: Cumulative Index to Nursing and Allied Health Literature
UN: United Nations
UNOCHA: United Nations Office for the Coordination of Humanitarian Affairs
UNICEF: United Nations International Children's Emergency Fund
NICE: National Institute for Health and Care Excellence
CT: Computed tomography
MRI: Magnetic resonance imaging
CCS: Country Capacity Survey

## References

[1] Wang Y, Wang J. Modelling and prediction of global non-communicable diseases. BMC Public Health. 2020;20(1):822. 32487173 10.1186/s12889-020-08890-4 PMC7268487

[2] World Health Organisation. Global cancer burden growing, amidst mounting need for services. https://www.imperial.ac.uk/media/imperial-college/administration-and-support-services/library/public/IMPP10650-College-Vancouver-Guide-230822-WEB.pdf. [Accessed 1st Jun 2024]. PMC11115397 38438207

[3] Sung H, Ferlay J, Siegel RL, Laversanne M, Soerjomataram I, Jemal A, et al. Global cancer statistics 2020: GLOBOCAN estimates of incidence and mortality worldwide for 36 cancers in 185 countries. CA Cancer J Clin. 2021;7(3):209–49. 10.3322/caac.21660 33538338

[4] United Nations Department of Economic and Social Affairs. World Population Prospects 2019. 2019. https://population.un.org/wpp/. Cited 2021 Apr 3.

[5] International Agency for Research on Cancer. Cancer Tomorrow. 2020. Available from: https://gco.iarc.fr/tomorrow/en/dataviz/bars?mode=population. Cited 2021 3 Apr 2021.

[6] World Health Organization. Cancer prevention and control in the context of an integrated approach. 2017.

[7] Jawad M, Vamos EP, Najim M, Roberts B, Millett C. Impact of armed conflict on cardiovascular disease risk: a systematic review. Heart. 2019;105(18):1388–94. https://heart.bmj.com/lookup/doi/10.1136/heartjnl-2018-314459. Cited 2021 May 7. 10.1136/heartjnl-2018-314459 31138670

[8] Fouad FM, Sparrow A, Tarakji A, Alameddine M, El-Jardali F, Coutts AP et al. Health workers and the weaponisation of health care in Syria: a preliminary inquiry for The Lancet–American University of Beirut Commission on Syria. Lancet. 2017;390(10111):2516–26. https://www.thelancet.com/journals/lancet/article/PIIS0140-6736(17)30741-9/fulltext?elsca1=tlpr#.YJWJSjXaToI.mendeley. Cited 2021 May 7. 10.1016/S0140-6736(17)30741-9 28314568

[9] Hanna TP, King WD, Thibodeau S, Jalink M, Paulin GA, Harvey-Jones E et al. Mortality due to cancer treatment delay: systematic review and meta-analysis. BMJ. 2020;371:m4087. https://www.bmj.com/content/371/bmj.m4087. 10.1136/bmj.m4087 PMC7610021 33148535

[10] Kutluk T, Koç M, Öner İ, Babalıoğlu İ, Kirazlı M, Aydın S et al. Cancer among syrian refugees living in Konya Province, Turkey. Conflict and Health. 2022;16(1):3. https://pubmed.ncbi.nlm.nih.gov/35101060/. 10.1186/s13031-022-00434-4 PMC8805424 35101060

[11] Al-Abdulla O, Sonsuz AA, Alaref M, Albakor B, Kauhanen J. The impact of humanitarian aid on financial toxicity among cancer patients in Northwest Syria. 2024;24(641). https://bmchealthservres.biomedcentral.com/articles/. 10.1186/s12913-024-11077-x PMC11102167 38762456

[12] Human Rights Watch. Syria: Events of 2019. 2019. https://www.hrw.org/world-report/2019/country-chapters/syria#. Cited 2021 Apr 3.

[13] UNOCHA, About OCHA. Syria. 2020. https://www.unocha.org/syrian-arab-republic/about-ocha-syria. Cited 2021 Apr 3.

[14] Physicians for Human Rights. PHR Statement on Attack on Al-Atareb Surgical Hospital, Northwest Syria. 2021. https://phr.org/news/phr-statement-on-attack-on-al-atareb-surgical-hospital-northwest-syria/. Cited 2021 May 21.

[15] Alhames S, Hsu A, Rustam F, Kassar R, Shihade M, Almhanna K. Esophageal cancer in Aleppo, Syria 2010–2020: a rare cancer in a war zone. Ann Transl Med. 2020;8(17):1105. 33145324 10.21037/atm-20-4474 PMC7575931

[16] Hopkins N. More than 80% of UN aid convoys in Syria blocked or delayed. The Guardian. 2016; https://www.theguardian.com/world/2016/sep/30/syria-un-aid-convoys-more-than-four-fifths-blocked-delayed-september.

[17] Abbara A. COVID-19 Exposes Weaknesses in Syria’s Fragmented and War-Torn Health System. MERIP. 2020. https://merip.org/2020/12/covid-19-exposes-weaknesses-in-syrias-fragmented-and-war-torn-health-system/. Accessed 2021 Apr 3.

[18] Nahhat F, Doyya M, Zabad K, Abo Laban T, Najjar H, Saifo M, Badin F. Breast cancer quality of care in Syria: screening, diagnosis, and staging. BMC Cancer. 2023;23(1234). https://bmccancer.biomedcentral.com/articles/10.1186/s12885-023-11740-2#. 10.1186/s12885-023-11740-2 PMC10722692 38097985

[19] Cancer Research UK Early Diagnosis Data Hub. Survival and Incidence by Stage at Diagnosis: Breast. https://crukcancerintelligence.shinyapps.io/EarlyDiagnosis/. [Accessed 1st Jun 2024].

[20] Kherallah M, Alahfez T, Sahloul Z, Eddin KD, Jamil G. Health care in Syria before and during the crisis. Avicenna J Med. 2012;2(3):51–3. https://pubmed.ncbi.nlm.nih.gov/23826546. 10.4103/2231-0770.102275 PMC3697421 23826546

[21] The World Bank. Current Health Expenditure (% of GDP). https://data.worldbank.org/indicator/SH.XPD.CHEX.GD.ZS?most_recent_value_desc=true [Accessed 1st May 2021].

[22] UK Aid. Protecting Healthcare in Syria. 2018. https://assets.publishing.service.gov.uk/media/5ba11d8ae5274a55a85179cd/Research_Report_-_Protection_of_Syrian_Health_Workers__August_2018.pdf . [Accessed 1st May 2021].

[23] Manachi M, Chatty E, Sulaiman S, Fahed Z. General Oncology Care in Syria. General Oncology Care in Syria. In: Al-Shamsi, H.O., Abu-Gheida, I.H., Iqbal, F., Al-Awadhi, A, editors Cancer in the Arab World. Singapore: Springer; 2022. P.265–284. 10.1007/978-981-16-7945-2_17.

[24] Marzouk M, Kelley M, Fadhil I, Slama S, Longuere K-S, Ariana P et al. If I have a cancer, it is not my fault I am a refugee: A qualitative study with expert stakeholders on cancer care management for Syrian refugees in Jordan. PLoS One. 2019;14(9). https://www.ncbi.nlm.nih.gov/pmc/articles/PMC6764666/. 10.1371/journal.pone.0222496 PMC6764666 31560701

[25] Page MJ, McKenzie JE, Bossuyt PM, Boutron I, Hoffmann TC, Mulrow CD et al. The PRISMA 2020 statement: an updated guideline for reporting systematic reviews. BMJ. 2021;372:n71. http://www.bmj.com/content/372/bmj.n71.abstract. 10.1136/bmj.n71 PMC8005924 33782057

[26] Penchansky R, Thomas JW. The Concept of Access: Definition and Relationship to Consumer Satisfaction. Med Care. 1981;19(2):127–40. http://www.jstor.org/stable/3764310. 10.1097/00005650-198102000-00001 7206846

[27] Wells G, Shea B, O’Connell D, Peterson J, Welch V, Losos M et al. The Newcastle-Ottawa Scale (NOS) for assessing the quality of nonrandomised studies in meta-analyses [abstract]. 3rd Symposium on Systematic Reviews: Beyond the Basics; 2000 Jul 3–5; Oxford, UK. 2000:15. https://academic.oup.com/jpubhealth/article/42/3/e299/5557733.

[28] CASP. Critical Appraisal Checklists. https://casp-uk.net/casp-tools-checklists/ [Accessed 3rd Jun 2024].

[29] Joanna Briggs Institute. Critical Appraisal Tools. https://jbi.global/critical-appraisal-tools [Accessed 3rd Jun 2024].

[30] Chattopadhyay A. Cross-Nation Comparison of Oral Cancer in the Eastern Mediterranean Region: an Ecological Overview. *Curr Oral Heal Reports*. 2015;2(3 PG-129–136):129–36. Available from: https://www.scopus.com/inward/record.uri?eid=2-s2.0-85100651195&doi=10.1007%2Fs40496-015-0058-7&partnerID=40&md5=9d8b7fc3da7073ac88b1fc9f8fde1216.

[31] Sadeghi M, Ghoncheh M, Mohammadian-Hafshejani A, Gandomani HS, Rafiemanesh H, Salehiniya H. Incidence and Mortality of Testicular Cancer and Relationships with Development in Asia. Asian Pac J Cancer Prev. 2016;17(9):4251–7. http://ovidsp.ovid.com/ovidweb.cgi?T=JS&PAGE=reference&D=med13&NEWS=N&AN=27797227. 27797227

[32] Ghoncheh M, Mohammadian M, Mohammadian-Hafshejani A, Salehiniya H. The Incidence and Mortality of Colorectal Cancer and Its Relationship With the Human Development Index in Asia. Ann Glob Heal. 2016;82(5):726–37. https://www.sciencedirect.com/science/article/pii/S2214999616307792. 10.1016/j.aogh.2016.10.004 28283123

[33] Kulhánová I, Bray F, Fadhil I, Al-Zahrani AS, El-Basmy A, Anwar WA et al. Profile of cancer in the Eastern Mediterranean region: The need for action. Cancer Epidemiol. 2017;47:125–32. http://www.elsevier.com. 10.1016/j.canep.2017.01.009 28268206

[34] Fitzmaurice C, Alsharif U, El Bcheraoui C, Khalil I, Charara R, Moradi-Lakeh M, et al. Burden of cancer in the Eastern Mediterranean Region, 2005–2015: findings from the global burden of Disease 2015 study. Int J Public Health. 2018;63(1):151–64. 28776254 10.1007/s00038-017-0999-9 PMC5973975

[35] Goodarzi E, Momenabadi V, Seraji M, Naemi H, Khazaei Z. Incidence and mortality of breast cancer and human development index: an updated study on the Asian population in 2018. Med Sci. 2020;24(102):623–31.

[36] Kulhánová I, Znaor A, Shield KD, Arnold M, Vignat JJ, Charafeddine M et al. Proportion of cancers attributable to major lifestyle and environmental risk factors in the Eastern Mediterranean region. Int J Cancer. 2020;146(3):646–56. http://onlinelibrary.wiley.com/journal/10.1002/(ISSN)1097-0215. 10.1002/ijc.32284 30882889

[37] Al-Hasan A, Murad R, Zaid KK, Al-Daoud J, Zaid KK, Al Hasan A et al. Epidemiological characteristics of retinoblastoma in children attending Almouassat University Hospital, Damascus, Syria, 2012–2016. Asian Pac J Cancer Prev. 2017;18(2):421–4. http://ovidsp.ovid.com/ovidweb.cgi?T=JS&PAGE=reference&D=medp&NEWS=N&AN=28345824. 10.22034/APJCP.2017.18.2.421 PMC5454737 28345824

[38] Kakaje A, Alhalabi MM, Ghareeb A, Karam B, Mansour B, Zahra B et al. Rates and trends of childhood acute lymphoblastic leukaemia: an epidemiology study. Sci Rep. 2020;10(4):6756. http://ovidsp.ovid.com/ovidweb.cgi?T=JS&PAGE=reference&D=emexc&NEWS=N&AN=631598956. 10.1038/s41598-020-63528-0 PMC7174306 32317670

[39] Salamoon M. Treatment of soft tissue sarcoma: results from the drug shortage era. J Hematol Diabetes. 2017;1(101):1–5.

[40] Rajeh N, Manchi M, Suliman S, Fahed Z, Barali M, Kalta M et al. Surviving stem cell transplantation program in war time in Syria. Blood. 2013;122(21):5525. http://bloodjournal.hematologylibrary.org/content/122/21/5525.abstract?sid=9cfea33c-c9b1-4d8e-ae51-ff9525e75be0.

[41] Sahloul E, Salem R, Alrez W, Alkarim T, Sukari A, Maziak W et al. Cancer Care at Times of Crisis and War: The Syrian Example. J Glob Oncol. 2016;3(4):338–45. 10.1200/JGO.2016.006189. 10.1200/JGO.2016.006189 PMC5560458 28831442

[42] Patenaude A, Fawaz O. A. P. Psychological challenges faced when fighting childhood cancer during wartime in Syria. Psychooncology. 2017;26(3):31. http://ovidsp.ovid.com/ovidweb.cgi?T=JS&PAGE=reference&D=emed18&NEWS=N&AN=617903491.

[43] Benbrahim Z, Mula-Hussain L, Al-Shamsi HO, El Saghir N, Al Asiri M, Al Bahrani B, et al. National approaches to managing cancer care: responses of countries in the MENA region to the COVID-19 pandemic. Ecancermedicalscience. 2021;15:1189. 33889198 10.3332/ecancer.2021.1189 PMC8043675

[44] Abdul-Sater Z, Menassa M, El Achi N, Abdul-Khalek RA, Abu-Sittal G, Mukherji D. Strengthening capacity for cancer research in conflict settings: key informant insights from the Middle East. Ecancermedicalscience. 2020;14:1153. 33574898 10.3332/ecancer.2020.1153 PMC7864685

[45] Bruni L, Serrano B, Roura E, Alemany L, Cowan M, Herrero R et al. Cervical cancer screening programmes and age-specific coverage estimates for 202 countries and territories worldwide: a review and synthetic analysis. Lancet Global Health. 2022;10(8): E1115-E1127. https://www.thelancet.com/journals/langlo/article/PIIS2214-109X(22)00241-8/fulltext#sec1. 10.1016/S2214-109X(22)00241-8 PMC9296658 35839811

[46] Hanafi I, Alsalkini M, Husein S, Salamoon M. The delay of breast cancer diagnosis and management during the Syrian war. Cancer Epidemiology. 2023;82:102290. https://www.sciencedirect.com/science/article/abs/pii/S1877782122001953. 10.1016/j.canep.2022.102290 36384074

[47] Atassi B, Tse G, Mkhallalati H, Debel J, Jemmo A, Khalil M et al. Cancer Diagnoses during Active Conflict: Experience from a Cancer Program in Northwest Syria. Avicenna J Med. 2022;12(4):157–161. https://www.ncbi.nlm.nih.gov/pmc/articles/PMC9771609/. 10.1055/s-0042-1755331 PMC9771609 36570430

[48] Harfouch RM, Alkhaier Z, Ismail S, Youssef A, Alhasan AA, Bouali F et al. Epidemiology and risk factors of colorectal cancer in Syria: a single-center retrospective study. European Review for Medical and Pharmacological Sciences. 2022;26:4654–4658. https://www.europeanreview.org/wp/wp-content/uploads/4654-4658.pdf. 10.26355/eurrev_202207_29187 35856355

[49] Baddour F, Al-Mahmoud A. Geographical Distribution of Cancer Patients Undergoing Radiotherapy in Different Regions of Syria. Tishreen University Journal. Health Sciences Series. 2021;43(1):87–103. https://journal.tishreen.edu.sy/index.php/hlthscnc/article/view/10404/9978.

[50] Burnett-Cargill A, Hamze M. Out of Sight: Obstacles in accessing breast cancer screening and treatment for women in Northwest Syria (NWS). Relief International and Syrian American Medical Society. 2023. https://www.sams-usa.net/wp-content/uploads/2023/05/Out-of-sight_FINAL.pdf.

[51] Faris G, Mouhamed M, Jerf F, Al, Khder N, Alnakry E, Salamoon M. Rapid Assessment of Cancer Management Care in Syria. 2016. https://reliefweb.int/report/syrian-arab-republic/rapid-assessment-cancer-management-care-syria-december-2016-enar#:~:text=In%20coordination%20with%20WHO%20Headquarters,to%20address%20the%20gaps%20in.

[52] World Health Organization Regional Office for the Eastern Mediterranean. Assessing national capacity for the prevention and control of noncommunicable diseases: report of the 2017 country capacity survey in the Eastern Mediterranean Region. 2017.

[53] World Health Organization. Cancer Country Profile: Syrian Arab Republic. 2020.

[54] World Health Organization. HeRAMS Annual Report: Public Hospitals in the Syrian Arab Republic. 2020.

[55] Amozorrutia-Alegría V, Bravo-Ortiz JC, Vázquez-Viveros J, Campos-Campos L, Mejía-Aranguré M, Juárez-Ocaña S, et al. Epidemiological characteristics of retinoblastoma in children attending the Mexican Social Security Institute in Mexico City, 1990-94. Paediatr Perinat Epidemiol. 2002;16(4):370–4. 12445155 10.1046/j.1365-3016.2002.t01-1-00442.x

[56] Pirouzian A, Mesfer S, Al Katan H, Maktabi AMY, Karoui M, Asghar N, et al. Retinoblastoma (rb) in Saudi Arabia- Fifteen Year Retrospective Comparative Review of a Registry: 1983–1997 vs. 1998–2007 at King Khaled Eye specialist hospital. Invest Ophthalmol Vis Sci. 2014;55(13):3081.24736053

[57] Lustosa de Sousa DW, de Almeida Ferreira FV, Cavalcante Félix FH, de Oliveira Lopes MV. Acute lymphoblastic leukemia in children and adolescents: prognostic factors and analysis of survival. Rev Bras Hematol Hemoter. 2015;37(4):223–9. 26190424 10.1016/j.bjhh.2015.03.009 PMC4519710

[58] Al-Mulla NA, Chandra P, Khattab M, Madanat F, Vossough P, Torfa E, et al. Childhood acute lymphoblastic leukemia in the Middle East and neighboring countries: a prospective multi-institutional international collaborative study (CALLME1) by the Middle East Childhood Cancer Alliance (MECCA). Pediatr Blood Cancer. 2014;61(8):1403–10. 24648275 10.1002/pbc.25031

[59] Lederer EM. UN warns that 90% of Syrians are below the poverty line, while millions face cuts in food aid. https://apnews.com/article/syria-humanitarian-aid-funding-crossborder-russia-5d28da9aa4d55b8c0f24563f69d8b5a0# [Accessed 4th Jun 2024].

[60] World Health Organization. Eastern Mediterranean (EMRO) Factsheet. 2021. https://gco.iarc.fr/today/data/factsheets/populations/993-who-east-mediterranean-emro-fact-sheets.pdf.

[61] Mehrvar A, Faranoush M, Asl AAH, Tashvighi M, Fazeli MA, Mehrvar N et al. Epidemiological Features of Childhood Acute Leukemia at MAHAK’s Pediatric Cancer Treatment and Research Center (MPCTRC), Tehran, Iran. Basic Clin Cancer Res. 2015;7(1 SE-Original Articles). https://bccr.tums.ac.ir/index.php/bccrj/article/view/168.

[62] Benitez Fuentes JD, Morgan E, de Luna Aguilar A, et al. Global stage distribution of breast Cancer at diagnosis: a systematic review and Meta-analysis. JAMA Oncol. 2024;10(1):71–8. 10.1001/jamaoncol.2023.4837. 37943547 10.1001/jamaoncol.2023.4837 PMC10636649

[63] Aburas R, Najeeb A, Baageel L, Mackey TK. The Syrian conflict: a case study of the challenges and acute need for medical humanitarian operations for women and children internally displaced persons. BMC Med. 2018;16(1):65. 10.1186/s12916-018-1041-7. 10.1186/s12916-018-1041-7 PMC5946430 29747641

[64] UNHCR. Burning trash to keep warm in Syria town cut off by war. 2016. https://www.unhcr.org/uk/news/latest/2016/11/58380bc24/burning-trash-keep-warm-syria-town-cut-war.html [Accessed 20th May 2021].

[65] Kakaje A, Alhalabi MM, Alyousbashi A, Ghareeb A, Hamid L, Al-Tammemi AB. Smoking habits and the influence of war on cigarette and shisha smoking in Syria. PLoS One. 2021;16(9):e0256829. https://www.ncbi.nlm.nih.gov/pmc/articles/PMC8412248/. 10.1371/journal.pone.0256829 PMC8412248 34473786

[66] El Saghir NS, Soto Pérez de Celis E, Fares JE, Sullivan R. Cancer Care for Refugees and Displaced Populations: Middle East Conflicts and Global Natural Disasters. Am Soc Clin Oncol Educ B. 2018;(38):433–40. 10.1200/EDBK_201365. 10.1200/EDBK_201365 30231320

[67] Abbara A, Rayes D, Fahham O, Alhiraki OA, Khalil M, Alomar A et al. Coronavirus 2019 and health systems affected by protracted conflict: The case of Syria. Int J Infect Dis. 2020;96:192–5. https://www.sciencedirect.com/science/article/pii/S1201971220303088. 10.1016/j.ijid.2020.05.003 PMC7205638 32389845

[68] Jawad M, Millett C, Sullivan R, Alturki F, Roberts B, Vamos EP. The impact of armed conflict on cancer among civilian populations in low- and middle-income countries: a systematic review. Ecancermedicalscience. 2020;14. https://ecancer.org/en/journal/article/1039-the-impact-of-armed-conflict-on-cancer-among-civilian-populations-in-low-and-middle-income-countries-a-systematic-review. 10.3332/ecancer.2020.1039 PMC7289611 32565892

[69] Mwaka AD, Wabinga HR, Mayanja-Kizza H. Mind the gaps: a qualitative study of perceptions of healthcare professionals on challenges and proposed remedies for cervical cancer help-seeking in post conflict northern Uganda. BMC Fam Pract. 2013;14:193. https://pubmed.ncbi.nlm.nih.gov/24341601. 10.1186/1471-2296-14-193 PMC3915559 24341601

[70] Witter S, Zou G, Diaconu K, Senesi RGB, Idriss A, Walley J et al. Opportunities and challenges for delivering non-communicable disease management and services in fragile and post-conflict settings: perceptions of policy-makers and health providers in Sierra Leone. Confl Health. 2020;14(1):3. 10.1186/s13031-019-0248-3. 10.1186/s13031-019-0248-3 PMC6945746 31921333

[71] Das M. Syrian cancer patients regain treatment access in Türkiye. Lancet Oncology. 2023;24(7):728. https://www.thelancet.com/journals/lanonc/article/PIIS1470-2045(23)00290-5/abstract. 10.1016/S1470-2045(23)00290-5 37331356

[72] Oxford Analytica Daily Brief. Syrian government forces may retake Damascus suburbs. Oxford Analytica. 2016. https://dailybrief.oxan.com/Analysis/DB216042/Syrian-government-forces-may-retake-Damascus-suburbs [Accessed 4th Jun 2024].

[73] Physicians for Human Rights. Aleppo Abandoned - A Case Study on Health Care in Syria. 2015. https://phr.org/our-work/resources/aleppo-abandoned/ [Accessed 4th Jun 2024].

[74] The World Bank. Physicians (per 1,000) - Syrian Arab Republic. 2021. https://data.worldbank.org/indicator/SH.MED.PHYS.ZS?contextual=default&locations=SY [Accessed 20th May 2021].

[75] The New Arab. Ceasefire deal sees Hayat Tahrir al-Sham take over Syria’s Idlib. The New Arab. 2019. https://english.alaraby.co.uk/english/news/2019/1/10/ceasefire-deal-sees-hts-take-over-syrias-idlib [Accessed 4th June 2024].

[76] Aji A. Bashar al-Assad fires his PM amid worsening economic crisis. The Age. https://www.theage.com.au/world/middle-east/bashar-al-assad-fires-his-pm-amid-worsening-economic-crisis-20200612-p55214.html . [Accessed 4th Jun 2024].

[77] Devi S. Economic crisis hits Lebanese health care. Lancet. 2020;395(10224):548. http://www.ncbi.nlm.nih.gov/pubmed/32087781. 10.1016/S0140-6736(20)30407-4 32087781

[78] Karak F, El, Rawadi E, Sawan J, Haddad FG. The impact of disasters on cancer care in Lebanon. Futur Oncol. 2021;17(6):629–31. 10.2217/fon-2020-0927. 10.2217/fon-2020-0927 33399027

[79] World Health Organization. Cancer Country Profile: Lebanon. WHO. https://www.who.int/cancer/country-profiles/LBN_2020.pdf?ua=1 [Accessed 20th May 2021].

[80] World Health Organization. WHO position paper on mammography screening. Geneva. 2014. https://www.ncbi.nlm.nih.gov/books/NBK269545/. 25642524

[81] World Health Organization. Comprehensive Cervical Cancer Control: A guide to essential practise – 2nd ed. Geneva. 2014. https://www.who.int/publications/i/item/9789241548953. 25642554

[82] Abdel-Razeq H, Attiga F, Mansour A. Cancer care in Jordan. Hematol Oncol Stem Cell Ther. 2015;8(2):64–70. http://europepmc.org/abstract/MED/25732671. 10.1016/j.hemonc.2015.02.001 25732671

[83] Boufkhed S, Harding R, Kutluk T, Husseini A, Pourghazian N, Shamieh O. What is the preparedness and Capacity of Palliative Care Services in Middle-Eastern and North African countries to respond to COVID-19? A Rapid Survey. J Pain Symptom Manage. 2021;61(2):e13–50. 33227380 10.1016/j.jpainsymman.2020.10.025 PMC7679234

[84] Human Rights Watch. *Syria: Events of 2020*. https://www.hrw.org/world-report/2021/country-chapters/syria [Accessed 20th May 2021].

[85] International Atomic Energy Agency. *PACT Mission to Syria.* https://www.iaea.org/newscenter/news/pact-mission-to-syria [Accessed 8th September 2024].

[86] Simaan S, Al Jerf F. Cancer in Syria (magnitude of the problem). International Journal of Cancer and Treatment. 2018;1(1):10–15. https://www.innovationinfo.org/articles/FRCM/IJCT-1-102.pdf.

[87] International Agency for Research on Cancer. Data and methods by country: Cancer incidence and mortality data sources and methods by country within region. https://gco.iarc.fr/today/en/data-sources-methods-by-country-detailed?tab=18 . [Accessed 8th Sept 2024].

[88] Fan KM, Rimal J, Zhang P, Johnson NW. Stark differences in cancer epidemiological data between GLOBOCAN and GBD: Emphasis on oral cancer and wider implications. eClinicalMedicine. 2022;54:101673. https://www.thelancet.com/journals/eclinm/article/PIIS2589-5370(22)00403-5/fulltext. 10.1016/j.eclinm.2022.101673 PMC9561675 36247925

[89] Critical Appraisal Skills Programme. Different Types of Bias In Research. https://casp-uk.net/news/different-types-of-research-bias/ [Accessed 8th Sept 2024].

[90] WHO. Global Essential Medicines. https://global.essentialmeds.org/dashboard/countries [Accessed 8th Sept 2024].

[91] International Agency for Research on Cancer. Frequently Asked Questions: Are the GLOBOCAN estimates for previous years. (e.g. 2018) still available online. https://gco.iarc.fr/today/en/about#faq [Accessed 8th Sept 2024].

